# Determinants of Fruit, Vegetable, and Dairy Consumption in a Sample of Schoolchildren, Northern Serbia, 2012

**DOI:** 10.5888/pcd10.130072

**Published:** 2013-10-31

**Authors:** Sanja Šumonja, Budimka Novaković

**Affiliations:** Author Affiliation: Budimka Novaković, University of Novi Sad, Novi Sad, Serbia.

## Abstract

**Introduction:**

Insufficient intake of fresh fruits, vegetables, and dairy products among children is a serious nutrition-related concern. The aim of our study was to determine behavioral and environmental factors that influence fruit, vegetable, and dairy consumption among Serbian schoolchildren.

**Methods:**

We used 24-hour recall to measure fruit, vegetable, and dairy intake of schoolchildren (N = 212) aged 8 to 11 years from 2 elementary schools in a local community in Serbia. We evaluated potential determinants of intake by using a 48-item questionnaire that asked about children’s behaviors, perceptions of others’ expectations and behaviors, reinforcement of children’s behaviors, and availability of fruits, vegetables, and dairy at home and school. Children completed written questionnaires during 1 school class under teacher supervision. Binary logistic regression was used to analyze determinants of fruit, vegetable, and dairy intake.

**Results:**

Negative predictors of fruit intake were expectations from teacher and parents (*P* <.001) to eat fruit and availability of fruit in school. Vegetable intake was positively related to paternal modeling behavior (*P* <.001) and availability of vegetables at home (*P* = .04). Dairy intake was positively influenced by parental reinforcement (*P* = .03).

**Conclusion:**

Various personal and environmental factors are associated with children’s intake of fruits, vegetables, and dairy. Interventions to promote fruit, vegetable, and dairy consumption in Serbian schoolchildren should focus on modeling and reinforcement by parents and teachers and increasing availability at school and at home.

## Introduction

According to research conducted in the United States, Europe, and Serbia, low intake of fruits, vegetables, and dairy among children is a serious nutrition-related concern (1–3). Inadequate eating habits in childhood may lead to iron-deficiency anemia, dental caries, and undernutrition (4). Low intake of fruit and vegetables has been associated with increased risk of overweight and obesity and subsequent chronic diseases, including cardiovascular disease and cancer (5). Sufficient intake of calcium from dairy foods (eg, milk, yogurt, cheese, milk-based desserts) is important in childhood because of the rapid growth experienced and its influence on genetically determined peak bone mass and obesity-related metabolic disorders (6). Prevalence of overweight and obesity among children is increasing in many countries (5). Approximately 20% of children in Europe are estimated to be overweight (2). According to the Institute for Public Health of Serbia, 18% of children in Serbia are overweight or obese (3).

Tracking eating habits from childhood to adulthood is important for creating interventions that aim to improve the eating habits of populations (7). Nutrition behavior in children is influenced by multiple personal, social, and environmental factors (8). Theories of behavior are useful for explaining the multiple influences on children’s nutrition behaviors (9). Two theories that have proved to be effective theoretical frameworks for studies aimed at increasing fruit and vegetable consumption among children — the integrated behavior model and social cognitive theory — were used as theoretical models of this study (9–11). The integrated behavior model explains behavior as a result of intention determined by attitude toward the behavior (feelings about behavior and behavioral beliefs), perceived norm (others’ expectation and others’ behavior), and personal agency (facilitators or constraints for performing behavior and self-efficacy about carrying out the behavior) (8). According to social cognitive theory, behavior is a result of dynamic and reciprocal interaction between personal factors (outcome expectations, self-efficacy, observational learning) and environmental factors (incentive motivation, facilitation) (9,10).

The relevance of each category of theoretical models may vary for different behaviors and for different populations (9), so defining the degree to which the nutrition behavior of a particular population is influenced by each category is important to create an effective intervention to influence that behavior in that population (9). The beginning of elementary school may be a transitional period for children’s dietary habits, as children spend less time with their parents and more time at school. Therefore, the aim of this study was to determine how strongly different personal and environmental factors are predictive of fruit, vegetable, and dairy intake in a sample of elementary schoolchildren in Serbia.

## Methods

Between February and May 2012, we collected data in this cross-sectional study through a self-report survey of a convenience sample of children aged 8 to 11 years in a local community in northern Serbia. We chose 2 schools of 5 elementary schools in the community and approached each school to obtain informed consent from school authorities to participate in the study. We selected 1 class from grades 2 through 5 from both schools. Children completed their questionnaires during 1 school class under teacher supervision. Before administering the questionnaires, we instructed teachers on how the questionnaires should be completed. Eligible children and their parents gave informed written consent to participate in the study. Research was approved by the ethics committee of the faculty of medicine of the University of Novi Sad.

We developed the questionnaire to meet the criteria of this study in 2 phases. In the first phase, we conducted an open-ended elicitation interview in a group setting with a sample of 30 children aged 8 to 11 years from both schools to identify relevant behavioral outcomes for, referents of, environmental facilitators of, and barriers to dietary intake (9). The interview included questions about participants’ feelings about performing the behavior, outcomes of performing the behavior, individuals who are in favor of or opposed to performing the behavior, and situational or environmental facilitators and barriers that make the behavior easy or difficult to perform (9). In the second phase, we developed the final survey instrument on the basis of previous research on determinants of fruit, vegetable, and food consumption in children and information obtained from the elicitation interviews (11–14). Two psychologists assessed whether the content of behavior was represented in the questionnaire by comparing questionnaire items with theories of behavior.

We used a 24-hour food-recall form to measure mean group intake and to identify the types of fruits, vegetables, and dairy foods eaten the day before the interview (15). Data about fruit, vegetable, and dairy intake was obtained in servings per day as recommended by the United States Department of Agriculture (USDA) (16,17). We assessed potential predictors of fruit, vegetable, and dairy intake by using a 48 item-questionnaire (fruits, 17 items; vegetables, 15 items; dairy, 16 items), which inquired about behavioral and environmental factors. Factors assessed were attitude toward the behavior (cognitive, 8 items [eg, “If I eat fruit/vegetable/dairy I will have more/will be . . .”] and affective, 3 items [“I like to eat fruit/vegetable/dairy because . . .”]), normative beliefs of others’ expectations (9 items, “I eat fruit/vegetable/dairy because my mother/father/teachers/friends tell[s] me to do it”), normative beliefs of others’ behavior (9 items, “My mother/father/teacher/friends eat[s] fruit/vegetable/dairy every day”), reinforcements (12 items, “I would eat more fruit/vegetable/dairy if . . .”), and availability of fruits, vegetables, and dairy foods at home and school (7 items, “I always have fruit/vegetable/dairy in home/at school”) (9,15). Participants chose yes if they agreed and no if they did not agree with each item. The questionnaire also included data about participants’ sex and age. Cronbach’s α coefficients ranged from 0.26 (7 items) for availability of fruit, vegetable, and dairy at home and school to 0.88 (12 items) for reinforcements.

We used SPSS version 18.0 (IBM Corporation, Armonk, New York) for data analyses. We conducted 3 separate binary logistic regressions to assess whether the investigated constructs were predictive of fruit, vegetable, and dairy intake in schoolchildren (18). The response options were recoded into a dichotomous variable for recommended number of servings for fruits, vegetables, and dairy (less than recommended = 0 vs recommended or more than recommended = 1). Intake of fruit, vegetable, and dairy was entered as dependent variable and theoretical model constructs were independent variables. We used χ^2^ test of independence to compare answers to questionnaire items according to sex and age (18). Children were stratified by age according to grade. *P* values less than .05 were considered to be significant.

## Results

We distributed 250 questionnaires, and 224 (89.6%) were returned. We excluded 12 questionnaires from analysis because they were incomplete; the total sample was 212 children (Table 1). Child mean age was 9 years (standard deviation = 1.07; range, 8–11). More than half of the participants (52.4%) consumed the recommended 1.5 or more servings of fruit per day, 17% consumed 2 or more servings of vegetables per day, and 11. 8% consumed 3 or more servings of dairy products per day. Girls (57.7%) were more likely to eat vegetables than boys (26.6%; χ^2^ = 11.96; *P* = .02)

**Table 1:** Sample Characteristics and Intake of Fruits, Vegetables, and Dairy, by Sex and Age, Schoolchildren in Northern Serbia, 2012

Characteristic	n (%)	Intake					
		Fruit		Vegetable		Dairy	
		Mean (SD)	RS^a^	Mean (SD)	RS^a^	Mean (SD)	RS^a^
**Total**	212 (100)	1.9 (1.01)	NA	1.6 (1.01)	NA	1.6 (1.00)	NA
**Sex**							
Male	90 (42.5)	1.9 (1.13)	1.5	1.2 (1.03)	1–2	1.4 (0-91)	2–3
Female	122 (57.5)	1.9 (1.09)	1.5	1.7 (1.02)	1.5–2.5	1.5 (0.91)	2–3
**Age, y (grade)^b^**							
8 (2nd)	52 (24.5)	1.5 (0.94)	1.5	1.1 (0.93)	1–1.5	1.5 (0.91)	2
9 (3rd)	59 (27.8)	2.4 (0.78)	1.5	1.7 (1.01)	2–2.5	1.9 (0.69)	3
10 (4th)	55 (25.9)	1.9 (1.06)	1.5	1.8 (0.91)	2–2.5	1.6 (0.91)	3
11 (5th)	41 (19.3)	1.9 (1.19)	1.5	1.6 (1.19)	2–2.5	1.4 (1.06)	3

Abbreviations: RS, recommended servings; SD, standard deviation; NA, not applicable.

a US Department of Agriculture’s recommendations for number of servings per day of fruit, vegetable, and dairy for children, according to age and sex (18).

b N = 207 (97.6%).

Most participants reported they like to eat fruits (44.8%), vegetables (39.5%), and dairy foods (38.4%) because these foods “make them feel strong and healthy.” Taste was the second most common reason for children’s preferences for fruits (36.5%), vegetables (31.8%), and dairy foods (36.7%). Girls (95.8%) reported stronger preferences for vegetables than boys (81.1%; χ^2^ = 7.08; *P* = .02).

The final logistic regression model accounted for 40.6% to 58.9% of variance for fruit intake, 42.9% to 59.1% for vegetable intake, and 37.3% to 50.5% for dairy intake. Children who reported consuming less than the recommended number of servings of fruit more often indicated their parents (*P* < .001) and teacher (*P* < .001) tell them to eat fruit and more often indicated they often have fruit for meals at school (*P* < .001) (Table 2). Positive predictors of vegetable intake were perceived paternal behavior (*P* < .001) and availability of vegetables at home (*P* = .04) (Table 2). Children who consumed less than recommended number of servings of dairy foods more often reported they would drink more milk if their parents drank milk (*P* = .03) (Table 2).

**Table 2:** Questionnaire Responses Predicting Participants’ Intake of Fruits, Vegetables, and Dairy, Schoolchildren in Northern Serbia, 2012

Theoretical Model Constructs (Questionnaire Items)	Yes, %	No, %	β	*P* Value^a^	OR (95% CI)
Fruits					
**Cognitive attitude**					
If I eat fruit I will have more energy.	97.6	2.4	NC	NC	NC
If I eat fruit I will always be hungry.	6.2	92.5	24.84	.99	6.13 (0)
If I eat fruit I will be thin.	17.9	78.3	1.21	.37	3.36 (0.24-47.41)
**Normative beliefs of others’ expectations**					
I eat fruit because my parents tell me to do it.	24.1	75.8	−4.09	<.001	0.02 (0-0.26)
I eat fruit because my teacher tells me to do it.	53.8	46.2	−4.23	<.001	0.15 (0-0.27)
I eat fruit because my friends tell me to do it.	9.9	90.1	−0.34	.84	0.71 (0.02-20.37)
**Normative beliefs of others’ behaviors**					
My mother eats fruit every day.	65.1	31.1	1.40	.31	4.07 (0.27-60.44)
My father eats fruit every day.	53.8	40.1	2.12	.15	8.32 (0.47-148.4)
My friends eat fruit every day.	46.7	43.9	1.23	.27	3.43 (0.38-31.12)
**Reinforcements**					
I would eat more fruit if my parents ate fruit.	21.2	78.8	1.45	.37	4.27 (0.18-100.84)
I would eat more fruit if my friends ate fruit.	17.1	82.9	−2.28	.29	0.10 (0-7.44)
I would eat more fruit if we could get fruit for meals at school.	55.7	44.3	2.07	.13	7.95 (0.55-114.45)
I would eat more fruit if I could buy fruit at school.	34.4	65.6	−0.41	.78	0.66 (0.04-11.86)
**Availability**					
We always have some fruit at home.	87.3	12.6	0.98	.51	2.67 (0.14-51.07)
We often have fruit for meals at school.	58.0	42.0	−2.07	<.001	0.15 (0.02-1.00)
I can buy fruit at school.	17.5	82.5	−5.61	.04	0 (0-0.89)
**Vegetables**					
**Cognitive attitude**					
If I eat vegetables I will have more energy.	97.5	2.4	NC	NC	NC
If I eat vegetables I will always be hungry.	6.2	92.5	−1.54	.46	0.21 (0-12.67)
If I eat vegetables I will be thin.	17.9	78.3	1.04	.36	2.84 (0.31-26.31)
**Normative beliefs of others’ expectations**					
I eat vegetables because my parents tell me to do it.	24.1	75.9	0.62	.61	1.87 (0.17-20.69)
I eat vegetables because my teacher tells me to do it.	53.8	46.2	−0.89	.34	0.41 (0.06-2.54)
I eat vegetables because my friends tell me to do it.	9.9	86.9	0.44	.81	1.55 (0.05-52.34)
**Normative beliefs of others’ behaviors**					
My mother eats vegetables every day.	67.5	32.5	−3.11	.06	0.04 (0-1.12)
My father eats vegetables every day.	63.2	36.8	4.30	<.001	74.1 (3.09-177.67)
My friends eat vegetables every day.	42.9	57.1	−1.41	.89	0.86 (0.10-7.27)
**Availability**					
We always have some vegetables for lunch at home.	66.5	33.5	1.65	.04	5.21 (1.03-26.30)
**Reinforcements**					
I would eat more vegetables if my parents ate vegetables.	21.	74.1	−3.15	.18	0.04 (0-4.31)
I would eat more vegetables if my friends ate vegetables.	17.0	77.8	−23.04	.99	0 (0)
I would eat more vegetables if we could get vegetables for meals at school.	39.2	55.2	0.86	.53	2.36 (0.16-35.31)
I would eat more vegetables if I could buy vegetables at school.	34.4	59.0	−2.16	.21	0.12 (0-3.27)
**Dairy Products**					
**Cognitive attitude**					
If I drink milk I will have strong bones and teeth.	94.8	3.2	NC	NC	NC
If I drink milk I will be fat.	2.5	92.5	NC	NC	NC
**Normative beliefs of others’ expectations**					
I drink milk because my parents tell me to do it.	17.0	82.0	−0.18	.87	0.84 (0.09-7.15)
I drink milk because my teacher tells me to do it.	35.4	64.6	1.59	.09	4.91 (0.76-31.51)
I drink milk because my friends tell me to do it.	6.1	93.8	−4.55	.09	0.01 (0-2.00)
**Normative beliefs of others’ behaviors**					
My mother drinks milk every day.	47.2	52.8	2.14	.05	8.56 (0.96-76.25)
My father drinks milk every day.	38.7	61.3	−1.42	.17	0.24 (0.03-1.86)
My friends drink milk every day.	43.9	47.2	0.37	.68	1.44 (0.26-8.14)
**Availability**					
We always have milk at home.	83.5	16.5	−0.73	.52	0.48 (0.05-4.60)
We often have milk for meal at school.	59.0	41.0	2.78	.77	0.09 (0.02-0.51)
I can buy milk at school.	16.0	84.0	3.20	.11	24.6 (0.46-302.14)
**Reinforcements**					
I would drink more milk if my parents drank milk.	22.2	72.6	−1.95	.03	0.14 (0.35-72.16)
I would drink more milk if my friends drank milk.	18.9	75.0	−0.72	.56	0.49 (0.04-5.70)
I would drink more milk if we could get milk for meals at school.	47.6	47.2	1.86	.13	6.43 (0.58-70.67)
I would drink more milk if I could buy milk at school.	33.0	61.3	−1.82	.18	0.16 (0.01-2.38)

Abbreviations: OR, odds ratio; CI, confidence interval; NC, not calculated.

a *P* < . 05 considered significant.

Significantly fewer 8-year-old children perceived that their mother (χ^2^ =14.3; *P* = .01), father (χ^2^ = 14.3; *P* = .01,) and friends (χ^2^ = 11.9; *P* = .02) eat fruit every day. They were also less likely to perceive that their mother (χ^2^ = 14.6; *P* = .01) and father (χ^2^ = 12.6; *P* = .01) eat vegetable every day. Significantly fewer 8-year-old children perceived they can buy fruit at school (χ^2^ = 10.8; *P* = .03). Availability of fruits (χ^2^ = 10.4; *P* = .03), vegetables (χ^2^ = 10.7; *P* = .03), and dairy (χ^2^ = 11.3; *P* = .02) at school was less important for children aged 8 years than for those aged 9 to 11 years as a reinforcement to consume these foods.

## Discussion

Similar to the results of other studies conducted in Serbia, the United States, and Europe (1–3), our results indicate that low intake of fruits, vegetables, and dairy among schoolchildren is a concern. In our study, children consumed on average 1.5 or more servings of fruit and more than 1 serving of vegetable and dairy per day. 

Nearly half of the children in our study, however, did not consume the recommended 1.5 servings of fruit per day. In the Pro Children study, the proportion of children who consumed more than 1 fruit daily varied from 15% (Norway) to 30% (Portugal) (19); the Youth Risk Behavior Surveillance survey showed that 64% of American children consume more than 1 fruit daily (1). Vegetable intake in our study was below USDA recommendations. Only 17% of children consumed 2 or more servings of vegetables. This finding is consistent with those of other studies indicating that inadequate vegetable intake is a greater nutrition-related concern than inadequate fruit intake (1,19). Nearly 12% of the children in our study met the USDA recommendation of 3 or more servings of dairy products per day. A study conducted in several European countries suggested that calcium intake in European children is below recommendations. American studies show that a decrease in milk consumption is followed by an increase in sweetened beverage consumption (6). These trends may lead to a decrease in calcium intake and an increase in carbohydrate intake in children. In our study, boys consumed fewer vegetables and reported less preference for vegetables than did girls. Taste preference is the strongest mediator of sex differences in fruit and vegetable intake in children (20,21). Possible explanations for sex differences in preferences for fruits and vegetables may be a tendency among boys to eat energy-dense food or a greater influence of social desirability on responses among girls because diet is more important for girls (22).

Participants in our study reported “health” as the most important reason for liking to eat fruits, vegetables, and dairy foods; children may be aware of the benefits of fruits, vegetables, and dairy foods on health, which may result in a tendency to give socially desirable answers. A study about nutrition knowledge and behavior conducted among Serbian schoolchildren and their parents indicated discrepancies in nutrition knowledge and behavior in children (23). Although children knew which foods are “healthier” (eg, fruit juice, fruit) they were more likely to choose “unhealthy” foods (sugar-sweetened carbonated beverages, chocolate) (23). In our study, almost all children recognized the positive effects on health of consuming fruits, vegetables, and dairy foods. However, cognitive attitudes did not significantly influence children’s intake of these foods, which could indicate that children have declarative knowledge (ie, awareness of things) but lack procedural knowledge (ie, how to do things) (24). Although children may be aware of the benefits of consuming fruits, vegetables, and dairy foods, they may not know how many of these foods they should eat per day, because nutrition education is not part of elementary school curriculum in Serbia. Nutrition education and knowledge of the recommendations can influence eating habits in children (24,25).

Parents can influence development of children’s eating behavior through child-feeding practices and modeling behavior and by providing food in the home (20,21,26–28). Most children in our study perceived that fruits and vegetables are available at home, but just a little over half of them reported that their parents eat fruits and vegetables every day. The perceived norm of parental eating behavior is a significant influence on children’s intake of fruits, vegetables, and dairy foods (21,26–28). In our study, perceived parental eating behavior significantly influenced only children’s intake of vegetables. In line with previous research, our study showed that home availability significantly influenced vegetable intake in children (21,26,28). Children who perceived that their parents consumed fruits and vegetables every day were more likely to consume more vegetables. It is expected that home availability and parental behavior influence vegetable intake, because vegetables are often consumed for meals at home in the family environment in Serbia. Although most children reported that dairy foods are available at home, less than half of them reported that their parents drink milk every day. Parents as role models were perceived as significant reinforcement for children in this study to consume more dairy food. Children who consumed less dairy food were more likely to report that they would drink more milk if their parents drank milk.

Younger children in this study were less likely to report that their parents eat fruits and vegetables every day than older children. Depending on the actual intake of fruits and vegetables of parents, this result could be explained in 2 ways. It could be a consequence of either more or less critical observation of parental behavior in younger children. Perceptions among younger children about family food rules may be more important than perceptions among older children, because the behaviors of the family have more of an effect on eating behaviors on younger than on older children (29). One study found that perceptions of children’s fruit and vegetable intake significantly differed between parents and children (29). Further study into the relationship between parents’ and children’s perception about parental intake is needed to understand how children of different ages perceive parental nutrition behavior.

Children in our study perceived more pressure to eat fruits, vegetables, and dairy from teachers than from parents — perhaps because children spend much of their day at school or because data were collected by teachers. However, children who perceived that their parents and teachers tell them to eat fruit tended to eat less fruit. These results confirm that feeding practices that involve pressuring children to eat fruit may negatively influence fruit intake in school-aged children (21,26,28). We recommend further research on how different teaching styles can improve fruit, vegetable, and dairy intake in children..

Most children in our study reported low availability of fruits, vegetables, and dairy at school. Children who perceived that fruit is often served for meals or that it can be bought at school were less likely to consume the recommended number of servings of fruit. Schools that took part in this study do not offer or sell fruit for meals, but fruit can be bought in stores near the school. Children who do not consume fruit may not be interested in the ways they can obtain fruit when they are at school; therefore, they may have responded that fruit is available at school if it is possible to buy fruit in the store close to the school. Availability of fruits, vegetables, and dairy at school was the most important reinforcement for children to consume more these foods. These results confirm earlier findings that school is one of the most important environmental factors that affect eating habits in children (20,21,28).

Younger children were more likely than older children to perceive low availability of fruit at school. It is expected that older children perceive more availability than younger children, because they are more independent in choosing foods and they often look for meals outside school. Younger children usually bring to school meals that parents prepared for them, which may also explain why school availability is a more important reinforcement for older children to consume more fruits, vegetables, and dairy. Further research is necessary to explore determinants of fruit, vegetable and dairy intake in children of different ages.

There are several limitations of this study. Intake of fruits, vegetables, and dairy was assessed by one 24-hour recall, which does not allow conclusions about average intake of these foods. However, 24-hour recall is easy and fast to complete, can be administered to children without help from parents or caregivers, and correlates significantly to observed intakes (30). Our sample was small and included children aged 8 to 11 years from only 2 schools, so our results may not be generalizable to the entire population of school-aged children in Serbia. The cross-sectional design of the study does not allow conclusions about how much change in correlates is predictive of change in intake of fruits, vegetables, and dairy.

Our study indicated that personal and environmental factors are associated with fruit, vegetable, and dairy intake among schoolchildren in Serbia. Interventions to promote fruit, vegetable, and dairy consumption in Serbian schoolchildren should focus on modeling and reinforcement by parents and teachers and increasing availability of fruits, vegetables, and dairy at school and at home. Future studies should examine more determinants of intake of fruits, vegetables, and dairy by using different instruments for accessing dietary intake in a representative sample of schoolchildren.
